# Incorporating Molecular Tools into Early-Stage Clinical Trials

**DOI:** 10.1371/journal.pmed.0050021

**Published:** 2008-01-22

**Authors:** Robert J Weil

## Abstract

The author discusses the implications of a new phase I trial investigating the role of rapamycin in patients with glioblastoma.

During the past several decades, and with an accelerating pace in the past several years, a primary focus of cancer research and treatment has been the development and refinement of specific, biologically directed therapies [1,2]. A number of attractive targets have been identified, dissected, and validated molecularly and biochemically, including multiple members of the family of receptor tyrosine kinases [1,2]. These potent enzymes, frequently concentrated or overexpressed on the surface of cancer cells, phosphorylate target proteins, with varied and manifold effects on numerous downstream, intracellular signaling pathways, leading to profound alterations in transcription and translation, cell growth, differentiation, apoptosis, angiogenesis, and invasion and metastatic potential [1,2]. A number of small molecular inhibitors of these tyrosine kinases (TKs) have been developed in recent years. Imatinib, for example, has shown impressive activity in many patients with chronic myelogenous leukemia [3,4].

The success of imatinib in human trials, and subsequent work in the laboratory and the clinic in several other cancers in which TKs appear causative and where TK inhibitors (TKIs) appeared likely to be efficacious, spurred a great deal of interest and enthusiasm throughout the oncologic community [1,2]. This was equally true in neurooncology, where progress in treating patients with malignant gliomas, especially glioblastoma (GBM), has been slow and incremental [4–7].

## Treating Glioblastomas

**Figure 1:**
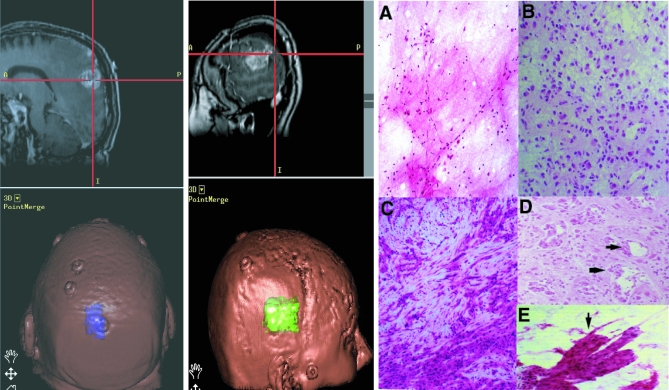
Clinicopathological Features of Glioblastoma Left, a sagittal (top), contrast-enhanced, T-1 weighted magnetic resonance (MR) image from a patient shows a left posterior parietal GBM, centered within the red cross during intra-operative navigation. The tumor is overlaid in purple on the skull (left, bottom); the several small discs seen on the surface of the scalp are used for intra-operative localization. Middle, a sagittal (top), contrast-enhanced, T-1 weighted MR image from a different patient shows a GBM within the right anterior parietal and posterior temporal lobes, represented in green on the bottom image. Right, histological variability of GBMs. A, normal paucicellular temporal lobe. B, typical, hypercellular GBM from one patient 50 years of age. C, excessive stromal proliferation within a separate portion of the same patient seen in B. D and E, areas of pronounced vascular proliferation (arrows) found throughout the specimen from a second patient, also 50 years of age, whose clinical presentation (headache and seizure) and tumor on MR imaging was nearly identical to that of the patient in B. The patient in B had little vascular proliferation compared to the patient depicted in D; conversely, patient D had no areas of stromal proliferation. Magnification in A–D, 200×; 400× in E. Hematoxylin and eosin staining.

GBM is an aggressive, primary tumor of the central nervous system [8]. Because of their intrinsic, infiltrative nature, GBMs follow a malignant clinical course. Classified as World Health Organization grade IV astrocytic tumors, GBMs have a pronounced mitotic activity, substantial tendency toward neoangiogenesis (microvascular proliferation), necrosis, and proliferative rates three to five times higher than grade III tumors, the anaplastic astrocytomas. The clinical behavior of GBMs is often mimicked by unusual pathological presentations, which gave rise to the old moniker of “glioblastoma multiforme” (Figure 1). Even with the survival advantage provided by the recently developed protocol of concurrent chemoradiation followed by adjuvant alkylating chemotherapy with temozolomide (the Stupp regimen), the prognosis of patients with GBM remains poor, with median overall survival in the range of 9–15 months and two-year survival rates of 26% in the most favorable subgroup [9].

Several common genetic alterations, such as *EGFR* (epidermal growth factor receptor) amplifications on chromosome 7p, as well as losses on 9p *(p16),* 10q (*PTEN,* or phosphatase and tensin homolog deleted on chromosome 10), and 17p *(p53)* have been identified in a significant proportion of patients with malignant gliomas (reviewed thoroughly in [8]). Two clinically recognized forms of GBM, de novo or primary and secondary or progression, have been identified clinically and recapitulated at the molecular genetic level [8]. In de novo or primary GBMs, *EGFR* gene amplifications, often combined with gene rearrangements that lead to a constitutively active, truncated receptor (the most common is EGFR_vIII_), occur in GBMs that generally express wild-type *p53* [8,10–16]. In secondary tumors, progression from a low-grade glioma to a GBM involves the serial accumulation of genetic alterations that inactivate tumor suppressor genes such as *p53, p16, Rb,* and *PTEN*, or activate oncogenes such as *MDM2* and *CDKs 4* and *6*; alterations in EGFR are less common or absent [8]. Frequently, loss of PTEN function is a common feature in both types of GBMs [8]. Response to chemotherapy may be modified by the level of expression of methyl guanine methyl transferase (MGMT) [9]. *MGMT* hypermethylation decreases production of MGMT, which leads to a diminished ability to repair DNA damage caused by an alkylating agent; presence of hypermethylated *MGMT* correlated with an approximately two-month improved median survival in patients treated with the Stupp regimen compared with those without hypermethylation [9]. However, promoter methylation analysis of *MGMT* is highly dependent on the tumor, collection method, specimen quality, and operator, and there is no standard alternative to the Stupp regimen in patients with intact *MGMT* [9].

**Linked Research Article**

This Research in Translation discusses the following new study published in *PLoS Medicine:*

Cloughesy TF, Yoshimoto K, Nghiemphu P, Brown K, Dang J, et al. (2008) Antitumor activity of rapamycin in patients with recurrent PTEN-deficient glioblastoma. PLoS Med 5: e8. doi:10.1371/journal.pmed.0050008

In a phase I trial Charles Sawyers and colleagues investigated the role of rapamycin in patients with PTEN-deficient glioblastoma.

The high incidence of EGFR overexpression, amplification, or coexpression of the truncated, constitutively active EGFR_VIII_ in GBMs raised expectations that TKIs of the EGFR, such as gefitinib or erlotinib, would have significant positive treatment effects, while minimizing toxicity compared to other therapies [1,2,8]. EGFR activates an intracellular TK that leads to a signal transduction cascade that enhances survival and infiltration of GBM cells in vitro [10–14]. Overexpression of EGFR correlates with increased cellular proliferation, tumorigenesis, decreased apoptosis, and a poorer prognosis and may be associated, as well, with radioresistance [12,14–16]. In GBM cell lines, TKIs suppress anchorage-independent growth, prevent proliferation, and enhance apoptosis [5,7,8].

While the inhibition of EGFR with TKIs showed promise preclinically, these inhibitors have subsequently shown only moderate activity as single agents in patients with GBM and other cancers. In one trial, 10 of 24 (42%) patients with recurrent or progressive GBM receiving erlotinib had a partial response or stable disease with a median time to progression of about 4.5 months—but the results of others have not been as favorable [5,6]. Analogous results were observed with EGFR antagonists in patients with non-small cell lung and pancreatic cancers [17,18]. Furthermore, Vogelbaum et al., for example, have observed that response to erlotinib is not determined by *EGFR* amplification status or EGFR overexpression [5]; patients with normal and elevated levels of EGFR were equally likely to have a clinical response.

**Figure 2:**
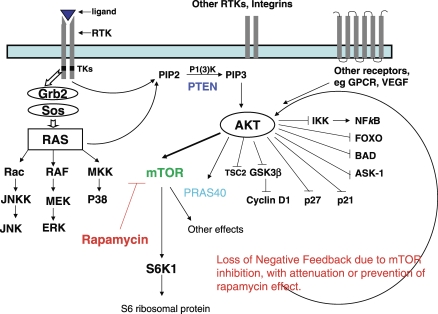
Cartoon Representation of Receptor Tyrosine Kinase and Phosphatidylinositol 3-Kinase (PI3K)/Akt/mTOR Pathways The cell surface is represented as a light blue rectangle and contains a variety of receptor tyrosine kinases, such as EGFR, insulin-like growth factor 1 (IGF-1R), and a variety or other receptors such as integrins, G-protein-coupled receptors (GPCRs), and the receptor for vascular endothelial growth factor (VEGF). Activation of the RTK by ligand (dark blue triangle) on the cell surface leads to dimerization of two receptors and phosphorylation at the tyrosine kinases, with intracellular activation of Grb2 and then Sos. Canonical activation of Ras leads to downstream activation of Rad, Raf, and MKK (mitogen-activated protein kinase kinase). It also leads, directly and indirectly through Ras, to generation of 3′-phosphoinositides, with activation of Akt; PTEN opposes the function of PI3K by removing its 3′-phosphate groups. Akt acts on a number of molecules and processes, both by activation (arrowheads) and by inhibition (lines with cross hatches), as indicated to the right of the figure. For our purposes, Akt directly activates mTOR, which is present in two complexes, not depicted here: TORC1 (mTOR bound to Raptor, whose substrates include S6K1 and PRAS40 and which is inhibited by rapamycin and its analogues) and TORC2 (mTOR bound to Rictor). mTOR activates S6K1, as shown, an effect inhibited by rapamycin (in red). As Cloughesy et al. demonstrate, however, this effect may be more complex than previously appreciated, since loss of mTOR activity by rapamycin blockade initiates a loss of negative feedback control on Akt, which may enhance its other growth-promoting effects. Definitions: ASK-1, apoptosis signal-regulating kinase, involved in regulating progression to apoptosis; BAD, the Bcl2 antagonist of cell death, involved in regulating progression to apoptosis; FoxO, forkhead box, involved in transcription and proliferation; GSK3β, glycogen synthase kinase 3-beta, involved in cell metabolism and growth; IKK, IκB kinase; NFκB, nuclear factor κB; PIP2, phosphatidylinositol-3,4-biphosphate; PIP3, phosphatidylinositol-3,4,5-triphosphate; TSC2, tuberous sclerosis complex 2.

Meanwhile, Haas-Kogan et al. and Mellinghoff et al. suggested that EGFR status and the activation status of some direct and indirect EGFR pathway components together play a role in the response to therapy in that fraction of patients (9%–18%) who respond favorably to erlotinib [19,20]. For example, coexpression of EGFR_VIII_ and PTEN was the most favorable molecular marker of response (six of seven patients who responded and were tested, from the nine patients out of 49 who had an objective treatment response) in the study by Mellinghoff et al. at the University of California, Los Angeles (UCLA) [19]. By contrast, none of the responders expressed EGFR_VIII_ in the study by Haas-Kogan et al., although overall elevated levels of EGFR and low or absent phosphorylated Akt levels were favorable predictors of response [20]. This has been confirmed by several groups who have returned to the laboratory to dissect the molecular mechanisms in vitro and animal preclinical models: in GBM cells with low PTEN expression levels, inhibition of the mammalian target of rapamycin, a downstream target of the phosphatidylinositol 3-kinase (PI3K) pathway through Akt (Figure 2), showed substantial efficacy [21–31].

## A New Study of Rapamycin for Recurrent GBM

These findings spurred the UCLA group to design an important, molecularly focused clinical study, published in this issue of *PLoS Medicine* [32], to analyze the effect of rapamycin in a subset of patients with recurrent GBM in whom activity of the tumor suppressor PTEN was absent. The study design, which is outlined in their Figure 1 [32], is a “treat-biopsy-treat” paradigm, in which *only* patients with the appropriate molecular features are selected to receive a targeted biological agent. In this case, patients who were known to have PTEN loss at the time of initial resection were chosen, after recurrence of tumor following standard treatment (surgery, radiation, and temozolomide), for inclusion in the study. Patients (*n* = 15) were treated for approximately one week with single-agent rapamycin, underwent resection, then resumed therapy and continued it until it was determined that tumor had recurred (time-to-progression).

**Figure 3:**
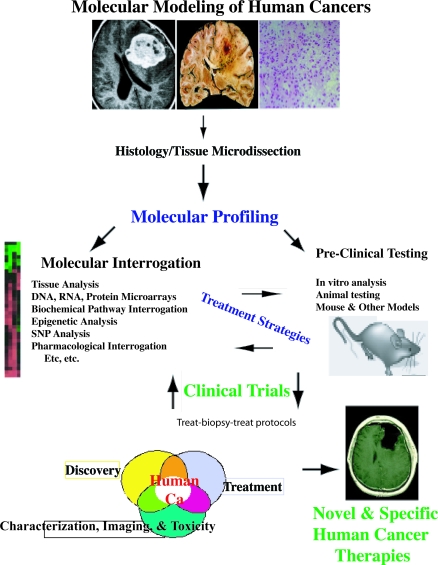
A Schematic Representation of the Potential for Novel Molecular Modeling of Human Cancer Therapy One potential paradigm is illustrated. Other methods and paradigms are possible.

A variety of well-designed molecular studies were conducted, including determination of serum and intratumoral concentration of rapamycin; markers of proliferation (Ki-67 labeling); assessment of the impact of mammalian target of rapamycin (mTOR) inhibition as measured by activation status of downstream targets of mTOR, including phospho-S6; and feedback loop inhibition of AKT (see Figure 4A in [32]). In seven of 14 patients (50%), suppression of mTOR correlated directly with inhibition of tumor cell proliferation, although in several other cases (non-responders), adequate intratumoral concentrations of rapamycin did not translate unequivocally into mTOR inhibition. In other words, the probability of response was greatest in patients with the greatest degree of mTOR inhibition ([32], Figure 3).

This illustrates the importance of performing these studies directly in humans, since preclinical data had failed to suggest that the levels of rapamycin used would be a problem. This failure is likely due to several features such as (1) the use in preclinical settings of higher doses (for greater saturation) than may be tolerable in humans; (2) factors such as the blood–brain and blood–tumor barriers, which influence pharmacodynamic bioavailability; (3) variability in vascularization and necrosis within the tumor; and (4) other host factors (see Figure 1). Thus, even in a carefully chosen cohort of patients with the molecular features that predict response, only 50% responded. Why?

An interesting and unexpected molecular feature appears to be responsible, at least in part: inhibition of mTOR led to feedback loop activation of Akt (depicted in Figure 2). This disinhibition appears to explain the diminished response rate, especially through activation of the downstream Akt target PRAS40. Genetic investigation of the factors associated with PRAS40 induction during mTOR inhibition identified amplification of *EGFR, MDM2,* and *PDGFRA* as more common in the non-responder subgroup, a finding not predicted from preclinical work.

Unpredictable results such as this have recently been echoed in three important studies, in which it has been shown in advanced solid epithelial malignancies, such as lung cancer and gliomas, that activation of multiple signaling pathways, as well as alteration of their feedback mechanisms, are common features and that successful treatment strategies must account for these novel characteristics of the neoplastic state [2,33–35]. Thus, use of combination therapy (for example, rapamycin plus a TKI or a TKI plus an inhibitor of Akt), as Cloughesy et al. suggest, or more permissive and less specific TKIs that work on several activation pathways (see Figure 2), as Arbiser advocates, is more likely to be successful when applied to specific subgroups of patients identified carefully along the lines described in the study published here [32]; see also the review of Arbiser [2].

**Five Key Papers in the Field**

**Engelman et al., 2007** [33] This paper shows how amplification of the *MET* oncogenes leads to enhancement of signaling through a receptor not generally activated in lung cancer.

**Furnari et al., 2007** [8] A comprehensive review of the molecular biology of the malignant gliomas.

**Kummar et al., 2007** [37] A cogent review of the issues surrounding careful interrogation of novel therapeutics. Essential reading.

**Stommel et al., 2007** [34] This paper, along with [33], illustrates the synergy between multiple receptor kinases and the potential need for more promiscuous TKIs, perhaps in combination with other signaling inhibitors such as rapamycin.

**Mellinghoff et al., 2005** [19] An elegant study that illustrates the dilemma of PTEN and Akt activation with the successes and failures associated with EGFR blockade in patients with GBM.

## Towards Individualized Therapy

The ultimate goal of most oncologists is to tailor therapy that takes into account—and exploits—the individual tumor's unique biological features. While individualized therapy may be some years in the future, the work of Cloughesy et al. [32], and that of many others, is pointing a way toward rational design of therapy for stratified groups of patients who share common molecular features [2,35–37]. Therefore, one strategy in designing the next generation of clinical trials in oncology must be to address both the known interactions, and, as Cloughesy et al. have done here, interrogate clinical studies and tissues at an early stage to identify genetic and biochemical features that distinguish responders and non-responders so that both types of patients receive optimal therapy (Figure 3).

This is likely to be an iterative process, which, as technology advances and neural network/machine-learning processes become integrated into clinical care, is likely to allow cancer researchers and clinicians to reach toward the holy grail of individualized therapy in the not-so-distant future [36]. It is also a method that has recently gained attention from both the United States Food and Drug Administration and the National Cancer Institute, with respect to rapid drug development timelines in cancer therapeutics and for tissue bio-repositories, with emphasis on what are being called “Phase 0 trials” [37]. The work of Cloughesy and his colleagues helps point the way.

## Abbreviations

EGFR: epidermal growth factor receptor
GBM: glioblastoma
MGMT: methyl guanine methyl transferase
MR: magnetic resonance
mTOR: mammalian target of rapamycin
PI3K: phosphatidylinositol 3-kinase
PTEN: phosphatase and tensin homolog deleted on chromosome 10
TK: tyrosine kinase
TKI: tyrosine kinase inhibitor
UCLA: University of California, Los Angeles
